# Cancer biomarkers for targeted therapy

**DOI:** 10.1186/s40364-019-0178-7

**Published:** 2019-11-15

**Authors:** Delong Liu

**Affiliations:** 1 New York Medical College, Valhalla, NY 10595 USA; 2 The First Affiliated Hospital of Zhengzhou University, Zhengzhou, 450052 China

**Keywords:** Biomarker, Tumor-associated antigen, BiTE, Antibody-drug conjugate, CAR-T

## Abstract

Tumor-associated antigens (TAA) or cancer biomarkers are major targets for cancer therapies. Antibody- based agents targeting the cancer biomarkers include monoclonal antibodies (MoAbs), radiolabeled MoAbs, bispecific T cell engagers, and antibody-drug conjugates. Antibodies targeting CD19, CD20, CD22, CD30, CD33, CD38, CD79B and SLAMF7 are in clinical applications for hematological malignancies. CD123, CLL-1, B cell maturation antigen, and CD138 are targets for cancer immunotherapeutic agents, including the chimeric antigen receptor - engineered T cells. Immune checkpoint inhibitors (ICIs) against PD-1, PD-L1, and CTLA-4 have led to the revolution of cancer immunotherapy. More ICIs targeting IDO, LAG3, TIM-3, TIGIT, SIGLECs, VISTA and CD47 are being explored. Small molecule inhibitors (SMIs) against tyrosine kinase oncoproteins such as BCR-ABL, JAK2, Bruton tyrosine kinase, FLT3, EGFR, ALK, HER2, VEGFR, FGFR, MEK, and MET have fundamentally changed the landscape of cancer therapy. SMIs against BCL-2, IDHs, BRAF, PI3 kinase, mTOR, PARP, and CDKs have become the mainstay in the treatment of a variety of cancer types. To reduce and avoid off-tumor toxicities, cancer-specific TAAs such as CD33 are being manufactured through systems biology approach. Search for novel biomarkers and new designs as well as delivery methods of targeted agents are fueling the next wave of advances in cancer therapy.

Tumor-associated antigens (TAA) or cancer biomarkers are major targets for cancer therapies. Antibody- based agents targeting cancer biomarkers include monoclonal antibodies (MoAbs), radiolabeled MoAbs, bispecific T cell engagers (BiTEs), and antibody-drug conjugates (ADCs) [1–6]. In the past few years, chimeric antigen receptor- engineered T cells (CAR -T) have become a major breakthrough in cancer immunotherapy [7–12]. In addition to the improvement in the design and manufacture of these targeted agents, search for new cancer biomarkers becomes equally critical. More agents targeting the following major biomarkers are rapidly migrating from bench to bedside for cancer therapy.

## CD19, the most targeted biomarker

CD19 is by far the most targeted biomarker for cancer immunotherapy [13]. One BiTE (blinatumomab) and two CAR-T products (tisagenlecleucel and axicabtagene ciloleucel) have been approved for clinical applications [2, 9, 14, 15]. More CD19 ADCs are in clinical trials, including coltuximab ravtansine (SAR3419), denintuzumab mafodotin (SGN-CD19A), loncastuximab tesirine (ADCT-402) [16–19]. It is worthwhile to note that CD19-targeted CAR-T, tisgenlecleucel, has shown activity against refractory /relapsed multiple myeloma in conjunction with high dose melphalan and autologous stem cell transplantation [20, 21].

## CD20, CD22, CD30, CD79b as targets for lymphoid malignancies

MoAbs against CD20 have been widely used for lymphoid malignancies [22, 23]. ADCs are increasingly used as chemoimmunotherapy. Four new ADCs have been approved for the treatment of lymphoid malignancies: brentuximab vedotin targeting CD30, inotuzumab ozogamicin and moxetumomab pasudotox targeting CD22, and polatuzumab vedotin targeting CD79b [3, 24–28]. More biomarkers are being targeted with ADCs or CAR- T cells. These biomarkers include CD25, CD37, CD56, CD70, CD74, and CD138 [29].

## CD33, CD123 and CLL-1 as targets for myeloid malignancies

Gemtuzumab ozogamicin (GO) is an ADC against CD33 that is widely expressed on myeloid cells [30]. GO has been approved for newly diagnosed as well as refractory /relapsed (RR) acute myeloid leukemia (AML) [31]. GO may be used as a single agent or in combination with chemotherapy regimens [32–34]. In addition, several novel ADCs targeting CD33 are under clinical development. These include vadastuximab talirine (SGN-CD33A), IMGN779, and AVE9633 (huMy9-6-DM4) [35–37].

ADCs targeting CD123, such as IMGN632 and SGN-CD123A, are being tested in clinical trials [38–41]. Further development of SGN-123A was however terminated due to safety concerns.

BiTE and ADCs targeting CLL-1 are currently undergoing preclinical or early clinical investigations for AML [42, 43]. CLL-1 - targeted CAR- T cells are in clinical trials for AML therapy [44, 45].

## Immune checkpoints for targeted immunotherapy

Immune checkpoint inhibitors (ICIs) against PD-1, PD-L1 and CTLA-4 have led to a fundamental paradigm shift in cancer immunotherapy [46–50]. One particular difference of ICIs from conventional chemotherapy is that the ICIs target immune cells instead of cancer cells and aim to modulate tumor microenvironment, leading to restoration of suppressed cancer immunity [51, 52]. More biomarkers of immune checkpoints including IDO, LAG3, TIM-3, TIGIT, SIGLECs, VISTA and CD47 are fueling the development of targeted agents [51, 53–59].

## B cell maturation antigen (BCMA) -targeted multiple myeloma therapy

BCMA is expressed in normal B cells, MM cells and malignant B cells [60–62]. Several CAR-T cell products targeting BCMA are in advanced clinical development for multiple myeloma (MM), including bb2121, LCAR-B38M, JCARH125, MCARH171, P-BCMA-101, CT053, and CT103A [63, 64]. In a recent report of a phase I study, 33 patients received bb2121 with an overall response rate (ORR) of 85% [65]. Sixteen patients were negative for minimal residue disease (MRD). LCAR-B38M is also in late stage clinical development. This CAR-T product contains a CAR targeting two BCMA epitopes [66, 67]. In a recent report of the LEGEND-2 trial, 57 patients who received infusion of LCAR-B38M CAR-T cells had an ORR = PR or better) of 88% [67]. In addition, BCMA is being targeted with BiTE and ADCs [68–71].

CS1 glycoprotein antigen (SLAMF7) is expressed on NK cells and MM cells. Elotuzumab is a MoAb that has been approved for RRMM therapy [72, 73]. CAR-T cells targeting SLAMF7 and light chains are in active development for therapy of RRMM [63, 64].

## Biomarkers for solid tumor immunotherapy

CD133-targeted CAR T cells have been used for solid tumors including cholangiocarcinoma [74–76]. Mesothelin- targeted CAR-T cells have been reported in mesothelioma, lung cancer, breast cancer, gastric cancer and pancreatic cancer [77, 78]. EGFR, HER2, and claudin18.2 are favorite targets for solid tumor immunotherapy including CAR-T and MoAbs [79–82]. T cell receptor- engineered T cells against AFP and MAGE-A1 have been reported for immunotherapy of solid tumors [83–88].

## Tyrosine kinase biomarkers as targets of small molecule inhibitors

Started with the BCR-ABL tyrosine kinase inhibitor (TKI), imatinib [89, 90], TKIs have become the mainstay of targeted oral agents for many cancer types [91–94]. Inhibitors of BCR-ABL, JAK2, FLT3, Bruton tyrosine kinase have led to a paradigm shift in the management of leukemias [95–100]. TKIs targeting a variety of tyrosine kinase oncoproteins, such as EGFR, ALK, HER2, FGFR, VEGFR, RET, MET, MEK have markedly changed the therapeutic landscape of such cancers as non-small cell lung cancer, breast cancer, bladder cancer, liver cancer, and renal cell carcinoma [101–106].

## BCL-2, IDHs, BRAF, PI3 kinase, mTOR, PARP and CDKs as targets of small molecule inhibitors

Biomarkers of many non-tyrosine kinase oncoproteins are major targets also for cancer therapy. Inhibitors of BCL-2, isocitrate dehydrogenases (IDH1 and IDH2), PI3 kinase, BRAF, mTOR, PARP and CDK have vastly expanded the armamentarium against a variety of cancer types, such as leukemia, lymphoma, melanoma, breast cancer, and ovarian cancer [107–116].

## Engineering biomarkers through systems biology for cancer therapy

TAAs are frequently expressed in normal tissues, therefore are not truly cancer-specific. Great efforts are being focused on engineering TAAs to improve or confer better cancer-specificity. CD33 is a myeloid marker and the target of the GO ADC in AML. BiTE and CAR-T cells are also being studied for clinical therapy of AML [117, 118]. However, off-tumor toxicities due to expression of CD33 in normal hematopoietic cells restrict the clinical applications. When CD33 gene was knocked out from the human hematopoietic stem and progenitor cells (HSPC), CD33- targeting CAR-T cells specifically eliminated AML cells without myelotoxicity in recipients transplanted with CD33- null HSPCs [119]. Clinically, the similar approach using systems biology to engineer HSPCs has been tested in a HIV+ patient with highly refractory acute lymphoblastic leukemia (ALL) [120]. In this case, CCR-5 of a fully HLA-matched allogeneic normal donor HSPCs were ablated with CRISPR technology. The donor HSPCs with ablated CCR-5 were transplanted into the HIV+ patient. The ALL went into complete remission with persistence of CCR-5 negative hematopoiesis. This approach using systems biology and engineering opens up a new era of manufactured cancer-specific biomarkers for targeted cancer therapy. Search for novel biomarkers and new designs as well as delivery methods such as nano-technology of targeted agents are fueling the next wave of advances in cancer therapy.

## Data Availability

This is not applicable.

## Abbreviation

CAR: Chimeric antigen receptor
